# Supplementary material for: Leveraging Digital Technology to Support Pregnant and Early Parenting Women in Recovery from Addictive Substances: A Scoping Review
Source: Int J Environ Res Public Health. 2023 Mar 2;20(5):4457. doi: 10.3390/ijerph20054457 (PMC10002058; doi:10.3390/ijerph20054457)
Supplement: Supplementary file 1 [file ijerph-20-04457-s001.zip › ijerph-2130959-supplementary.pdf]

Table S1. Results Table.

Article	Type of Study	Type of Digital Technology	Target Population to Include Family	Maternal and/or Fetal Outcomes	Substance Targeted for Intervention
Abroms et al., 2017	Two group randomized controlled trial	Text messaging; Text4baby Quit4baby	Text4baby; Quit4 baby subscribers were eligible if they had a pregnancy due date > 8 weeks prior to sending the recruitment text message	Results obtained via phone survey 1, 3, and 6 months after study enrollment, and Cotinine levels obtained via saliva sample at the 3 month follow up. More subscribers in the intervention group (28.20%) reported not smoking in the past 7 days at 1 month as compared to subscribers in the control group (15.79%) (*p* = 0.01), and 35.20% of the intervention group and 22.67% of the control group reported not smoking in the past 7 days at 3 months (*p* = 0.01).	Smoking: nicotine targeted as identified through method of saliva testing; Cotinine levels obtained via saliva sample.
Cummins, et. al., 2016	Two group randomized controlled trial	Telephonic counseling plus self-help materials	Recruited from state Quitline. 1,173 participants-- 58% white, 21% black, 13% Hispanic, 2.5% Asian, 3.6% American Indian, and 2% other	Measures obtained on prolonged abstinence at the third trimester (about 29 weeks' gestation) and at 2 and 6 months postpartum; Abstinence rates were higher for the intervention than the control group at the end of pregnancy, including 30-day abstinence, 2 months postpartum, 90-day abstinence, and 6 months postpartum; Cotinine- level 7-day abstinence rates at the end of pregnancy supported the intervention effect (35.8% vs 22.5%, o0.001)	Nicotine targeted as identified through method of saliva testing; cannabis smoking was not mentioned as part of exclusionary criteria
Evans et. al., 2012	Randomized pilot evaluation study	Text4Baby Mobile Health Program	Participants (n=123) randomized to enroll in Text4baby and receive usual health care (intervention) or usual care only (control); 90 completed follow-up interview.	Significant effect of Text4Baby intervention exposure on increased agreement with the attitude statement "I am prepared to be a new mother" (OR = 2.73, CI = 1.04, 7.18, p = 0.042) between baseline and follow-up. For women with a high school education or greater, there was a significantly higher overall agreement to attitudes against alcohol consumption during pregnancy (OR = 2.80, CI = 1.13, 6.90, p = 0.026); significant improvement of attitudes toward alcohol consumption from baseline to follow-up (OR = 3.57, CI = 1.13 – 11.24, p = 0.029).	Alcohol
Evans et. al., 2014	Randomized controlled trial (Initial outcomes reported)	Text4Baby (intervention) versus usual care (control)	Pregnant women presenting for care at Madigan Army Medical Center in Tacoma, Washington (n=943)	**Note:** Adjusted and unadjusted logistic equation models were developed to assess intervention effects on measured outcomes. In unadjusted models, there was a significant effect of intervention exposure on belief in the importance of visiting a health care provider to be a healthy new mother (OR 1.52, 95% CI 1.01-2.31, P=.046) and in the health risks of alcohol during pregnancy (OR 2.06, 95% CI 1.00-4.31, P=.05). No behavioral effects of the intervention were observed in this analysis.	Alcohol, Smoking
Evans et. al., 2015	Randomized controlled trial (looking specifically at dose response effect of Text4Baby on behavioral outcomes from	Text4Baby	Pregnant women at Madigan Army Medical Center (n=943) completed a baseline survey, 48.7% (459/943) completed a 4-week follow-up survey, and 24.5% (231/943) completed a postpartum follow-up.	Reported descriptive statistics including dosage of text messages delivered. The main finding was a significant effect of high exposure to Text4Baby on self-reported alcohol consumption postpartum (OR 0.212, 95% CI 0.046-0.973, P=.046), as measured by the question "Since you found out about your pregnancy, have you consumed alcoholic beverages?" The Text4Baby participants also reported lower quantities of alcohol consumed postpartum.	Alcohol

Article	Type of Study	Type of Digital Technology	Target Population to Include Family	Maternal and/or Fetal Outcomes	Substance Targeted for Intervention
	Evan et al., 2014 study)				
Forray et. al., 2019	Secondary analysis from Martino et al., 2018 randomized controlled trial (conducted to evaluate the potential differential effects of Screening Brief Intervention and Referral to Treatment [SBIRT] versus usual care on substance use among different substance subgroups).	E-SBIRT	Pregnant and non-pregnant women were recruited from reproductive health clinics (n=439); **Note**: Eighty of the women who met criteria for use of a primary substance were pregnant	The number of pregnant women reporting secondary substance use was the following: cigarette (n=69), alcohol (n=14) and illicit drugs (n=36). Pregnant women were less likely to be in the alcohol secondary substance group compared to nonpregnant women. Cigarettes were the most frequently reported primary substance (n=251), followed by illicit drugs (n =137) and alcohol (n=51). For primary cigarette use the interaction between the linear effect of time and treatment was significant for SBIRT, but not e-SBIRT, suggesting greater reductions in cigarette use over the first 3 months following treatment with SBIRT compared to enhanced usual care (EUC). Results followed a similar pattern for primary illicit drug use among the e-SBIRT group in which the interaction of e-SBIRT treatment with linear time, and quadratic time were statistically significant suggesting greater reductions in illicit drug use with e-SBIRT versus EUC, which attenuated with time. Neither SBIRT nor e-SBIRT was associated with a significant reduction in days of alcohol use per month, as compared to EUC.	Cigarettes, Alcohol, Illicit drugs
Fujioka, et. al., 2012	Experimental evaluation study using questionnaires and carbon monoxide exhalation measurements	e-learning cessation smoking-support program accessed via cell phone with internet capability	Pregnant Japanese women in their first trimester of pregnancy (n=66) who were greater than 20 weeks' gestation and receiving regular obstetric check-ups	52 of 66 pregnant smokers from three settings began the program, and 48 of 52 eventually completed it. The achievement rate of non-smoking was 71.1%, and their carbon monoxide exhalation levels significantly decreased from 6.43 _ 4.5 ppm at the beginning to 0.7 _ 1.0 ppm in 1 month, to 0.29 _ 1.08 in 3 months (P < 0.001). These results suggest the effectiveness of the e-learning program.	Nicotine/Smoking
Guille, et al., 2020	Nonrandomized controlled trial	Telemedicine	Pregnant women who received perinatal opioid use disorder treatment in 4 outpatient obstetric practices by telemedicine or in person (n=98).	No statistically significant differences in rates of retention in treatment between women receiving opioid use disorder treatment via telemedicine versus in person (80.4% vs 92.7%). These findings were also apparent in newborns with neonatal abstinence syndrome (telemedicine: 45.4% vs in person: 63.2%).	Opioid Use Disorders
Harris & Reynolds, 2015	Two group randomized controlled trial	Web-based contingency management (CM) program and a telephone delivered cessation counseling program (SCHB)	Community sample of pregnant women who were active smokers and resided in rural Appalachia Ohio and Kentucky (n=17)	For CM, two of seven participants (28.57%) achieved abstinence, and three of 10 (30%) of those enrolled in SCHB were abstinent by late in pregnancy. Participants in CM attained abstinence more rapidly than those in SCHB. However, those in SCHB experienced less relapse to smoking, and a greater percentage of these participants reduced their smoking by at least 50%.	Nicotine/Smoking

Article	Type of Study	Type of Digital Technology	Target Population to Include Family	Maternal and/or Fetal Outcomes	Substance Targeted for Intervention
Herbec et. al., 2014	Pilot randomized controlled trial	Internet-based smoking cessation intervention	Pregnant adult smokers in the United Kingdom (n=200) who participated in an online intervention assessing the effectiveness of MumsQuit compared with an information-only website.	A difference of 8% was found in the 4-week continuous abstinent rates between MumsQuit (28.3%) and the control (20.8%); the odds ratio was 1.5 (95% CI = 0.8–2.9). The results did not change after adjusting for baseline characteristics or in the complete case analysis. There was no indication that the telephone follow-up inflated abstinence rate, as participants followed up by telephone reported still smoking more often than those responding online (69.2% vs. 50.0%). Compared with control participants, those in the MumsQuit group logged in more often, viewed more pages, and spent more time browsing the website	Nicotine/Smoking
Johnston, et al. 2019	Naturalistic, non-equivalent control group quasi-experimental design	Addiction-Comprehensive Health Enhancement Support System (A-CHESS) smart-phone application	Pregnant or parenting Appalachian women mandated into treatment (n=98)	Treatment service use: A-CHESS group averaged 780 service units compared with 343 for the comparison group; A-CHESS group stayed in treatment a mean of 410 vs 262 days for the comparison group	Opioids<br>Alcohol, Marijuana, Benzos, Barbiturates
Kamke, et. al., 2019	Observational study	Smokefree MOM is a mobile text messaging program that provides 24/7 tips, advice, and encouragement to help pregnant women who want to quit smoking	Users of SmokefreeMOM program (n=1288). Average age was 29 years. Of these, 65.81% (848/1288) were white, 16.04% (n = 207) were black, 8.86% (n=114) were Latina, and 9.29% (n= 120) were multiracial, American Indian/Alaska Native, Native Hawaiian Pacific Islander, or other; 82.68% (n= 1065) had some college education or less.	Response and abstinence rates were similar across race, ethnicity, and education. Point-prevalence abstinence was 14.51% (157/1082) on quit day, 3.51% (38/1082) at intervention end, and 1.99% (21/1053) at 1-month follow-up. Black users and those with a high school degree or less or some college education were less likely to drop out than White users or users with a bachelor's degree or higher.	Smoking cessation (no substance explicitly identified although nicotine is inferred in background and significance section)
Kurti et. al., 2020	Pilot study-to examine the efficacy of leveraging a smartphone app to deliver the financial incentives intervention remotely	Smart phone application	Pregnant women throughout the U.S. (n=60)	Seven-day point prevalence abstinence rates were greater in the incentives versus best practices arms at 7 days, 4 and 8 weeks postpartum; although not greater at 12 and 24 weeks postpartum likely due to the study being underpowered for discerning differences at the later assessments, especially 24-weeks postpartum which was three months after treatment completion.	Nicotine use via smoking
Liang, et al., 2021	Mixed-methods analysis of web posts.	Study focused on women posting in an online health community (OHC) with opioid use or misuse during pregnancy.	Pregnant women who are in an OHC who mentioned at least one opioid use disorder - related drug name. 200 Web posts by pregnant women with	The majority of pregnant women (150/200, 75.0%) in the OHC exhibited signs of misuse, and 62.5% (125/200) of the participants were either contemplating or pursuing dosage reduction. Self-managed withdrawal was more common (P<.001) than professional treatment among the population. A total of 5 themes of self-management support needs were	Opioids

Article	Type of Study	Type of Digital Technology	Target Population to Include Family	Maternal and/or Fetal Outcomes	Substance Targeted for Intervention
			opioid use were thematically and computationally analyzed.	identified as women sought information about the potential adverse effects of gestational opioid use, protocols for self-managed withdrawal, pain management safety during pregnancy, hospital policies and legal procedures related to child protection, and strategies for navigating offline support systems.	
Love et. al., 2016	Feasibility study using a single group repeated measures design (pre, post, 6-month follow-up). Data collected included standardized self-report measures, post-intervention focus groups and interviews, website usage reports,<br>and Google analytics.	Smartphones to access social media and gaming features (e.g., social sharing with anonymity, badges to incentivize skills practice, an accredited facilitator for support) to an evidenced-based parenting program, Triple P (Positive Parenting Program) Online.	High-risk parents (n=155) (e.g., 76% had a family annual income of less than $15,000; 41% had been incarcerated; 38% were in drug/alcohol treatment; and 24% had a child removed due to maltreatment). The ethnic groups most commonly identified<br>were Hispanic (66%) and African American (24%). Respondents were primarily mothers<br>(86%) from five community programs in Los Angeles.	Significant multivariate ANOVA time effects were found, demonstrating reductions in child behavioral problems, reduced lax/permissive and over-reactive parenting, and decreased parental stress. No effects were found for parental confidence, attributions, or depression and anxiety (which were in the normal range at baseline). Positive effects were maintained or improved at 6-month follow-up. The participants engaged in the online community and valued its flexibility, anonymity, and shared learning.	No specific substance targeted with intervention; however, authors reported that<br>59 participants (38.06%) were in a drug/alcohol treatment program.
Martino et al., 2018	Randomized controlled trial that allocated women to 3 groups:<br>e-Screening Brief Intervention and Referral to Treatment (e-SBIRT), clinician-delivered SBIRT (SBIRT), or an educational pamphlet plus existing usual care<br>(EUC; control condition)	e-SBIRT	Women (81.8% were non-pregnant) from two reproductive centers who smoked cigarettes or misused alcohol, illicit drugs, or prescription medication (n=439) were randomly allocated to electronic-delivered Screening, Brief Intervention and Referral to Treatment, (n = 143) (16.8% pregnant), clinician-delivered Screening, Brief Intervention and Referral to Treatment (n = 145) (18.6% pregnant) and in enhanced usual care (n = 151) (19.2% pregnant)	Pregnant women reported substance use, on average, about 17% fewer days per month than<br>did non-pregnant women. Three-way interactions between treatment, time and pregnancy status did not differ significantly in the effect of treatment over time between pregnant and non-pregnant women, although study was not powered to test this difference. Both e-SBIRT and SBIRT significantly reduced days of primary substance use over the follow-up period compared to EUC. Estimated decline in number of substance-use days were greatest in e-SBIRT group; SBIRT significantly decreased days of primary substance use among women in reproductive healthcare centers; neither resulted in more treatment use than enhanced usual care.	Nicotine, Alcohol, Cannabis, other illicit drugs

Article	Type of Study	Type of Digital Technology	Target Population to Include Family	Maternal and/or Fetal Outcomes	Substance Targeted for Intervention
Mauriello et. al., 2011	Feasibility study	Healthy pregnancy: Step by step is a computer-tailored intervention	Underserved pregnant women from federally qualified health centers (n=87)	Overall feasibility and acceptability of the program with pregnant women noting an increase in intention to make behavioral changes. Smoking cessation included as part of intervention that also addressed fruit and vegetable consumption and stress management.	Nicotine
Moreland et. al., 2021	Focused on program evaluation of the transition of screening and treatment for mental health and substance use disorder to remote platforms due to COVID- 19 pandemic.	Text-messaged based screening<br>Online screening	Pregnant and postpartum women at The Medical University of South Carolina's [MUSC] Women's Reproductive Behavioral Health Program; Note: total number of women receiving online or text based screenings not provided	Reported significant increase in the utilization of maternal mental health and substance use disorder screening and treatment services for pregnant and postpartum women.	Opioids
Naughton et al., 2017	Large pilot randomized controlled trial	Text messaging	Women aged 16 years and over, and less than 25 weeks pregnant (n= 407) who smoked at least five cigarettes daily. Two hundred and three women were randomized to intervention group and 204 were randomized to control group. Recruited from 16 antenatal clinics in England.	Seven smoking outcomes, including validated continuous abstinence from 4 weeks post-randomization until 36 weeks gestation.<br>There was some indication, although not conclusive, that a text-messaging program may increase cessation rates in pregnant smokers when provided alongside routine NHS cessation care.	Smoking
Ondersma et al, 2018	Randomized controlled trial; women were randomly assigned to either a time control condition or a single session, tailored, indirect, brief intervention.	computer-delivered, brief intervention designed for use with indirect screen-positive cases, seeking to motivate reductions in drug use	The Wayne Indirect Drug Use Screener (WIDUS)-positive postpartum women (n=500). Two hundred and fifty-two women were allocated to the intervention and 248 were allocated to the control; mostly African American and non-Hispanic	Of the 500 participants (252 intervention and 248 control), 36.1% of participants acknowledged drug use in the 3 months prior to pregnancy, but 89% tested positive at the 6-month follow-up. Participants rated the intervention as easy to use (4.9/5) and helpful (4.4/5). Analyses revealed no between-group differences in drug use (52% in the intervention group, vs. 53% among controls; OR 1.03). Exploratory analyses also showed that intervention effects were not moderated by baseline severity, WIDUS score, or readiness to change. Concluded no evidence of efficacy for an indirect, single-session, computer-delivered, brief intervention designed as a complement to indirect screening	Screened correlates to drug use instead of drug use itself
Ondersma et al, 2014	Randomized control trial	Electronic screening and brief intervention (e-SBI)	Postpartum, primarily low-income African American women (n=143) meeting	Note: 7-day point-prevalence abstinence at each follow-up point, per self-report (using a Timeline Follow-Back approach) and	Marijuana and alcohol mainly (small percentage of cocaine and opioid users)

Article	Type of Study	Type of Digital Technology	Target Population to Include Family	Maternal and/or Fetal Outcomes	Substance Targeted for Intervention
			criteria for drug use, were randomly assigned to either a tailored e-SBI or a time-matched control condition.	evidence of abstinence per toxicology tests (urine analysis at the 3-month follow-up, and urine plus hair analysis at the 6-month follow-up). Blinded follow-up evaluation 3- and 6-months following childbirth revealed strong effects for confirmed illicit drug use abstinence at the 3-month observation as did hair analysis at 6 months. Additional primary outcomes suggested small to moderate effect sizes in favor of the e-SBI, but did not reach significance. This result replicates previous findings but fails to show durable effects.	
Paterno et al, 2018	Pilot—Moms Supporting conducted with the aims of assessing the feasibility of using digital story telling (DST) with peer mentors and understanding the meaning and process of recovery from the perspective of women with lived experience of perinatal substance use disorder (SUD).	digital storytelling (Storycenter.org)	Women with a history of SUD who served as peer mentors (n=5)	The digital storytelling workshop process helped peer mentors make linkages between their past substance use experiences to their present work of recovery and fostered deep social connections between mentors through the shared experience. The workshop process also elicited a sense of hope among participants, which served as groundwork for developing advocacy-based efforts. Digital storytelling may be therapeutic for women in recovery and has the potential to be integrated into recovery programs to bolster hope and social support among participants.	Unclear which substances: presumably opioids or alcohol as stories indicated babies with neonatal abstinence syndrome
Paterno et al, 2019	Qualitative pilot study (narrative storytelling)	Digital storytelling (Storycenter.org) as a mechanism for understanding substance use and recovery from the perspective of women	Women with a history of SUD who served as peer mentors (n=5) explored role of peer mentor with lived experience of SUD during pregnancy	Three key themes emerged from participants as they reflected on their roles as peer mentors while engaging in the workshop process: (1) making linkages from past to present, (2) fostering deeper social connections among mentors in the workshop through shared experience, and (3) identifying spaces of hope and a sense of purpose.	Unclear which substances: presumably opioids or alcohol as stories indicated babies with neonatal abstinence syndrome
Pollak, et al., 2020	Randomized controlled trial	Theory-based messages throughout pregnancy to reduce relapses (1/2 sample).	Pregnant women (n=314) from 14 prenatal clinics. Women were between 10 and 30 weeks pregnant, smoked at least 100 cigarettes in their lifetime, smoked	Women in each experimental group quit smoking at the same rate (9%-12%). Women in this study also significantly reduced their smoking habits from the beginning to the end of the pregnancy, reduced from nine cigarettes to four. No fetal outcomes reported.	Nicotine/Smoking

Article	Type of Study	Type of Digital Technology	Target Population to Include Family	Maternal and/or Fetal Outcomes	Substance Targeted for Intervention
			three or more cigarettes every day in the prior 7 days, were current smokers, were not using nicotine replacement therapy (NRT) in the prior 7 days, willing to try to quit smoking, were enrolled in prenatal care, were aged 18 or older, and who spoke English.		
Pollick et. al., 2015	Mixed methods	Computerized brief intervention for Alcohol Use in Pregnancy (C-BIAP)	African American pregnant women (n=18) screened for not currently using alcohol, but at risk for alcohol use	The C-BIAP received high ratings of acceptability; qualitative feedback was also positive overall and suggested good acceptance of abstinence themes	Alcohol
Sadicario, et al., 2021	Program evaluation transition from in-person psychology trainee services to telepsychology.	Telepsychology	Women receiving substance use disorder treatment (n= not provided in article) at the women's and addictions program at Virginia Commonwealth University.	VCU providers were able to identify and address a substantial number of opportunities to implement telepsychology within the program during the COVID-19 Pandemic. The program addressed barriers to care access in creative ways, including the use of various technologies to meet patients' needs and comfort levels. No specific maternal or fetal outcomes were reported. Focus was telepsychology usage as service delivery and continued timely collaboration.	Licit and illicit substances including opioids
Sanguan et al. (2019)	Pilot Nonexperimental correlational design looking at substance use/post-traumatic stress disorder (PTSD) symptoms and parental bonding	Smartphone with ecological momentary assessment app that queried about PTSD symptoms, prenatal bonding, and substance use three times daily for 28 days	Pregnant women (n=33)	74% adherence with technology; moderate associations with PTSD symptoms and substance use; parental bonding modestly associated with substance use; concluded daily PTSD symptoms as a risk factor and less consistent support for prenatal bonding as a protective factor for prenatal substance use.	Most of sample reported cigarette and alcohol use; noted significant heroin, marijuana, amphetamine use; some reported benzodiazepines, cocaine
Tzilos et al., 2011	Randomized controlled trial	Computer-delivered brief intervention	Pregnant women who screened positive for risky alcohol use (n=50)	Ratings of intervention ease of use, helpfulness, and other factors were high (4.7–5.0 on a 1–5 scale). Participants in both conditions significantly decreased alcohol use at follow-up, with no group differences; however, birth weights for infants born to women in the intervention group were significantly higher ($p<0.05$, $d=0.62$)	Alcohol
van der Wulp, et. al., 2014	Cluster randomized trial	Computer-tailoring respondents received usual care from their midwife and 3 computer-tailored	Adult pregnant women < or = 12 weeks; cluster randomized to : health counseling (n=135), computer tailoring (n=116), and usual care (n=142)	Computer-tailoring respondents stopped using alcohol more often compared to usual care respondents 6 months after baseline (53/68, 78% vs 51/93, 55%; P=.04). Multilevel multiple linear regression analyses showed that computer-tailoring respondents (mean 0.35, SD 0.31 units per week) with average	Alcohol

Article	Type of Study	Type of Digital Technology	Target Population to Include Family	Maternal and/or Fetal Outcomes	Substance Targeted for Intervention
		feedback letters via the Internet. Usual care respondents received routine alcohol care from their midwife. After 3- and 6- months, the effect of the interventions on alcohol use was assessed.		(P=.007) or lower (P<.001) Alcohol use before pregnancy or with average (p=.03) or lower (p=.002) social support more strongly reduced their alcohol use 6 months after baseline compared to usual care respondents (mean 0.48, SD 0.54 units per week). Six months after baseline, 72% (62/86) of the health-counseling respondents had stopped using alcohol. This 17% difference with the usual care group was not significant.	
Wernette et al. (2018)	Pilot randomized controlled trial	Computerized Screening Brief Intervention and Referral to Treatment (SBIRT) single-session brief motivational intervention plus booster session addressing both substance use and sexually transmitted infection risk.	Pregnant women (n=50); who were primarily Latina or African American	Intervention acceptability ratings were high, ranging between 6.3 and 6.8 on a 1–7 scale. At the 4-month follow-up, participants in the intervention arm reported a significantly larger reduction (54%) in any marijuana or alcohol use compared with participants in the control group (16%) (p = 0.015); higher reduction in condomless vaginal sex at follow-up in the health checkup for expectant moms (HCEM) arm than control (27% vs. 5%), although this was not statistically significant (p = 0.127).	Alcohol, cannabis, cocaine and/or opiates
